# Genome Sequence of a SARS-CoV-2 Strain from a COVID-19 Clinical Sample from the Khagrachari District of Bangladesh

**DOI:** 10.1128/MRA.00189-21

**Published:** 2021-04-01

**Authors:** M. Imranul Hoq, Robiul Hasan Bhuiyan, M. Khondakar Raziur Rahman, Imam Hossen, Sajib Rudra, M. Arif Hossain, Shanta Paul, M. Omer Faruq, Mohammad Omar Faruque, H. M. Abdullah Al Masud

**Affiliations:** a Department of Microbiology, Faculty of Biological Sciences, University of Chittagong, Chattogram, Bangladesh; b Department of Biochemistry and Molecular Biology, Faculty of Biological Sciences, University of Chittagong, Chattogram, Bangladesh; c Department of Botany, Faculty of Biological Sciences, University of Chittagong, Chattogram, Bangladesh; DOE Joint Genome Institute

## Abstract

This study describes the genome sequence of a severe acute respiratory syndrome coronavirus 2 (SARS-CoV-2) strain detected in the nasopharyngeal swab sample of a coronavirus disease 2019 (COVID-19) patient from the southeastern Khagrachari District of Bangladesh.

## ANNOUNCEMENT

The ongoing pandemic of coronavirus disease 2019 (COVID-19), which was first reported in Wuhan, China, in December 2019 (1), is caused by severe acute respiratory syndrome coronavirus 2 (SARS-CoV-2), a *Betacoronavirus* in the *Coronaviridae* family (2). In Bangladesh, the first COVID-19 case was detected on 8 March 2020 (3). We report here the genome sequence of SARS-CoV-2 from a 43-year-old male from the Khagrachari District of Bangladesh, who was hospitalized with fever, joint ache, diarrhea and difficulty breathing, and tested positive for COVID-19 by reverse transcriptase PCR (RT-PCR) (4). All the protocols were approved by the Ethical Review Board of the University of Chittagong (reference no. CUBIO0001). Informed consent was obtained from the patient, and the sample was collected with the permission of the Directorate General of Health Services of the Government of Bangladesh.

Viral RNA was extracted from the nasopharyngeal swab using a PureLink viral DNA/RNA minikit (catalog no. 12280050; Thermo Fisher Scientific). cDNA synthesis and library preparation were carried out using an Illumina RNA prep with enrichment (L) tagmentation kit (catalog no. 20040537) combined with Illumina respiratory virus oligonucleotide panel v2 (catalog no. 20044311), and the prepared libraries were sequenced on an Illumina MiniSeq system in the paired-end format (read length, 74 bp) according to the manufacturer’s instructions. Duplicates and low-quality reads were removed, and coverage plots were created using Illumina DRAGEN RNA pathogen detection version 3.5.15.

**FIG 1:**
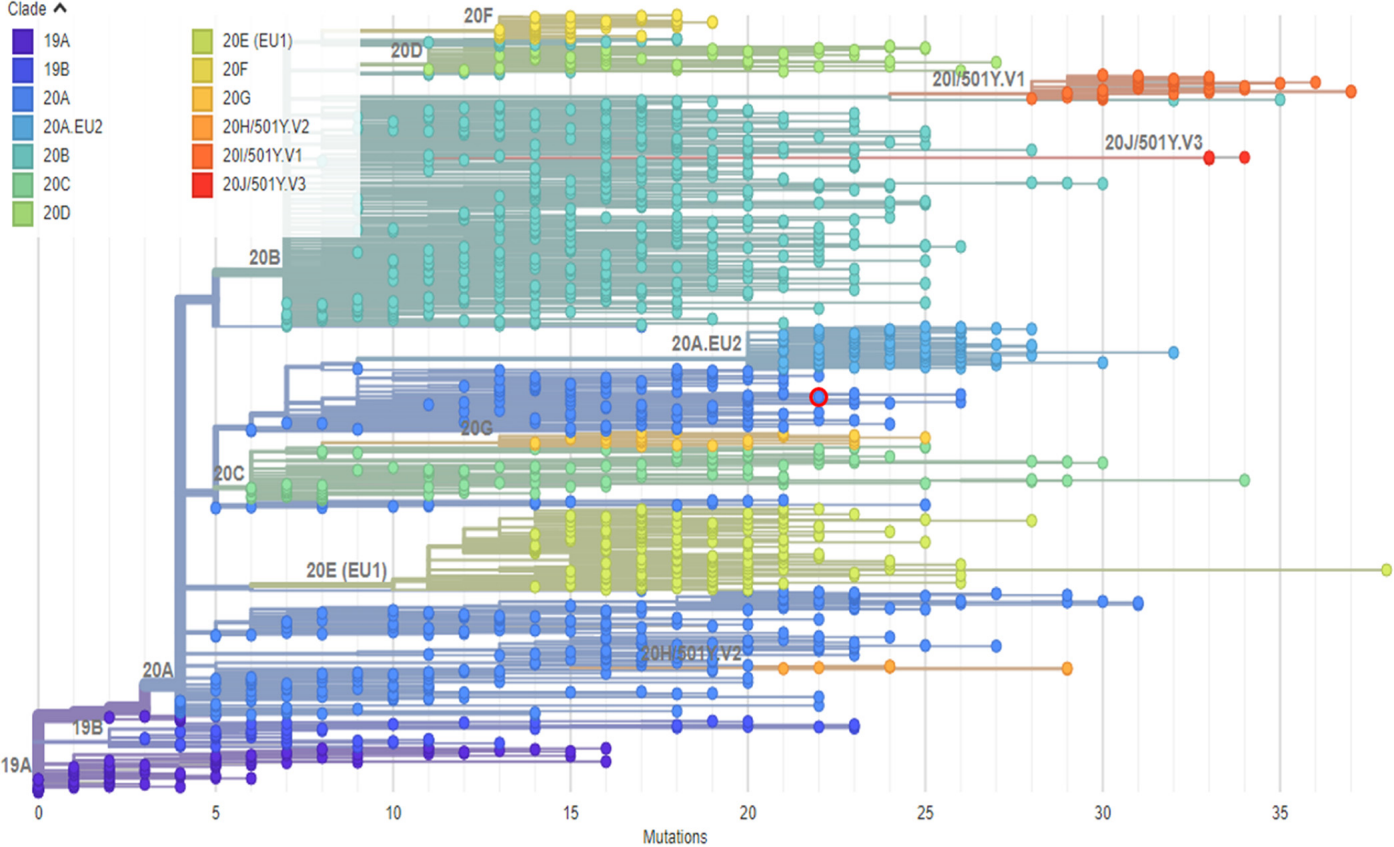
Phylogenetic tree of a SARS-CoV-2 strain from the Khagrachari District of Bangladesh. The tree was constructed on 19 February 2021 using the Nextclade^beta^ version 0.13.0 (clades.nextstrain.org), in which the red circle represents the position of hCoV-19/Bangladesh/CU-CTG-24/2020 (GISAID accession no. EPI_ISL_735496).

The library generated a total of 3,625,976 reads, of which 2,165,542 reads mapped to the reference sequence (GenBank accession no. MN908947.3) using human (hg38) and the Illumina respiratory virus panel, with the human control option of the DRAGEN software, and 1,709,268 reads were found unique after exclusion of the duplicates. The FASTQ data files were exported from the Illumina local run manager to the BaseSpace Sequence Hub; a consensus FASTA file was generated using k-mer analysis (GenBank accession no. NC_045512.2) of the DRAGEN software; and it was revealed that the genome of this strain has 29,856 bp which starts and ends at positions 7 and 29862, respectively, of the reference sequence (29,903 bp). The whole-genome comparison, using the DRAGEN software, of the strain revealed 99.85% identity, with the reference sequence having a mean coverage depth of 303×, whereas no indel was detected. The sequence displays a GC content of 38%. The consensus genome and related sample data were uploaded to the Global Initiative on Sharing All Influenza Data (GISAID) database (accession no. EPI_ISL_735496) on 25 December 2020 (5). Phylogenetic analysis using Nextclade^beta^ version 0.13.0 (clades.nextstrain.org) assigned the new genome to the SARS-CoV-2 clade 20A (Fig. 1) (6). According to the GISAID database basic local alignment search tool (BLAST), the genome shares the highest levels of similarity with sequences uncovered from Saudi Arabia (GISAID accession no. EPI_ISL_678004, EPI_ISL_513151, EPI_ISL_513149, EPI_ISL_437736, and EPI_ISL_437723) and India (GISAID accession no. EPI_ISL_1073009, EPI_ISL_1073011, EPI_ISL_1073010, and EPI_ISL_1073014) (5).

An analysis of the variations, using Genome Detective Virus Tools version 1.132 (7), indicates several changes in the sequence of this strain exhibiting 11 synonymous and 11 nonsynonymous mutations relative to the Wuhan-Hu-1 reference sequence (GenBank accession no. NC_045512.2) (Table 1). According to the GISAID database, 2 mutations, namely, P681H and V1122L, of the spike glycoprotein of this virus were rare among the SARS-CoV-2 strains recovered in Bangladesh (5). As of 19 February 2021, the mutation P681H was also observed in 5 other strains of SARS-CoV-2 recovered in Bangladesh (GISAID accession no. EPI_ISL_906098, EPI_ISL_906091, EPI_ISL_890237, EPI_ISL_890188, and EPI_ISL_774976), whereas the mutation V1122L is still unique in Bangladesh.

**TABLE 1:** Mutations observed in hCoV-19/Bangladesh/CU-CTG-24/2020 compared with SARS-CoV-2 isolate Wuhan-Hu-1^*a*^

Nucleotide position	Reference nucleotide	Mutated nucleotide	Gene	Amino acid change
241	C	T	5′-UTR^*b*^	Noncoding
1006	G	T	ORF1ab	K247N
1853	C	T	ORF1ab	None (synonymous mutation)
2836	C	T	ORF1ab	None (synonymous mutation)
3037	C	T	ORF1ab	None (synonymous mutation)
4331	C	T	ORF1ab	None (synonymous mutation)
4755	C	T	ORF1ab	P1497L
6472	C	T	ORF1ab	None (synonymous mutation)
7119	C	T	ORF1ab	S2285F
7247	T	C	ORF1ab	F2328L
14408	C	T	ORF1ab	P4715L
17056	A	G	ORF1ab	M5598V
18877	C	T	ORF1ab	None (synonymous mutation)
22444	C	T	S	None (synonymous mutation)
23403	A	G	S	D614G
23604	C	A	S	P681H
24130	C	T	S	None (synonymous mutation)
24926	G	T	S	V1122L
25563	G	T	ORF3a	Q57H
26735	C	T	M	None (synonymous mutation)
28854	C	T	N	S194L
29260	G	T	N	None (synonymous mutation)

a GenBank accession no. NC_045512.2.

b UTR, untranslated region.

### Data availability.

The sequence has been deposited in the GISAID database (accession no. EPI_ISL_735496) and GenBank (accession no. MW599343). The accession number for the raw sequence reads in the NCBI Sequence Read Archive (SRA) is SRR13718002. The BioProject and BioSample accession numbers are PRJNA701790 and SAMN17911680, respectively.
